# Challenging directions in pediatric diabetes - the place of oxidative stress and antioxidants in systemic decline

**DOI:** 10.3389/fphar.2024.1472670

**Published:** 2024-12-18

**Authors:** Vasile Valeriu Lupu, Ingrith Miron, Laura Mihaela Trandafir, Elena Jechel, Iuliana Magdalena Starcea, Ileana Ioniuc, Otilia Elena Frasinariu, Adriana Mocanu, Florin Dumitru Petrariu, Ciprian Danielescu, Alin Horatiu Nedelcu, Delia Lidia Salaru, Ninel Revenco, Ancuta Lupu

**Affiliations:** ^1^ Pediatrics, “Grigore T. Popa” University of Medicine and Pharmacy, Iasi, Romania; ^2^ Faculty of Medicine, “Grigore T. Popa” University of Medicine and Pharmacy, Iasi, Romania; ^3^ Pediatrics, “Nicolae Testemitanu” State University of Medicine and Pharmacy, Chisinau, Moldova

**Keywords:** diabetes, children, pro-oxidative status, antioxidants, nutrition

## Abstract

Diabetes is a complex condition with a rising global incidence, and its impact is equally evident in pediatric practice. Regardless of whether we are dealing with type 1 or type 2 diabetes, the development of complications following the onset of the disease is inevitable. Consequently, contemporary medicine must concentrate on understanding the pathophysiological mechanisms driving systemic decline and on finding ways to address them. We are particularly interested in the effects of oxidative stress on target cells and organs, such as pancreatic islets, the retina, kidneys, and the neurological or cardiovascular systems. Our goal is to explore, using the latest data from international scientific databases, the relationship between oxidative stress and the development or persistence of systemic damage associated with diabetes in children. Additionally, we highlight the beneficial roles of antioxidants such as vitamins, minerals, polyphenols, and other bioactive molecules; in mitigating the pathogenic cascade, detailing how they intervene and their bioactive properties. As a result, our study provides a comprehensive exploration of the key aspects of the oxidative stress-antioxidants-pediatric diabetes triad, expanding understanding of their significance in various systemic diseases.

## 1 Introduction

Diabetes occupies a leading place in the list of chronic pathologies with an increasing incidence rate. Comparatively analyzing the pre-pandemic and pandemic period, an interconnection was observed between respiratory infection with coronavirus and autoimmune pathologies (e.g., diabetes, systemic lupus erythematosus) (Fotea et al., 2023; Lupu et al., 2023a). D'Souza D. et al. observed the escalation of the number of newly diagnosed cases of diabetes (type 1 and 2) in the COVID-19 pandemic. In particular, in the case of type 1 diabetes, a numerical increase of more than ½ of newly diagnosed cases was observed in 2020 (the first pandemic wave) compared to 2019 (32%000 versus 19%000). The incident rate was 1.14 times higher in the first year and 1.27 times higher in the second year after the onset of the pandemic. A similar trend has been reported in type 2 diabetes. The precipitating factors of the association are the localization of the SARS-CoV-2 ACE2 entry receptor at the level of insulin-producing β cells, the switch to a sedentary lifestyle and psychological stress (D'Souza et al., 2023). Consequently, following the model above, we can state that the two entities (diabetes and acute respiratory infection with coronavirus) have a common physiopathological point represented by oxidative stress (Fotea et al., 2023). Previous data are now validated by studies on the positive impact of a nutrient/antioxidant balance on immune system activity, reducing inflammation and oxidative stress (Delmastro and Piganelli, 2011; Chernyak et al., 2020; Iddir et al., 2020).

Therefore, taking note of the international research trend, we consider that the early recognition of diabetic manifestations, the screening of populations at risk and the maximum therapeutic management (pharmacological and non-pharmacological) must be desired in an adequate patient care. This narrative review focuses on the implications of oxidative stress (framed by us as the “Cinderella of diabetic pathogenesis”) on the systemic evolution of patients with diabetes. Additionally, we discuss the way in which various dietary antioxidant substances (whether or not they have pharmacological similarities) intervene in the oxidative dynamics, emphasizing at the same time their main dietary sources.

## 2 State of the art

Diabetes in children tends to have a more severe onset than in adults, resulting in a much higher mortality rate. Consequently, type I diabetes is one of the most common chronic diseases of childhood. With an increasing incidence, especially under the age of 5, type I diabetes occurs in 1/350 children under the age of 18. Furthermore, type II diabetes currently describes an increase in frequency parallel to that of childhood obesity. A peak of the incidence is therefore noted in the post-puberty period (15–19 years). One of the rarest and most aggressive forms of diabetes is neonatal, whose diagnosis and management protocol is important to know (Chisnoiu et al., 2023; Calabria, 2022; Koren and Levitsky, 2021). To this is added the risk of acute/chronic complications (metabolic/organic), doubled by the possible damage to bone development and integrity (Vanderniet et al., 2022; Ahmad E. et al., 2022; El Amrousy et al., 2021). The most feared acute complication is ketoacidosis, characterized by the smell of ketones, dehydration, abdominal pain, Kussmaul breathing, vomiting, coma, altered mental status (Los and Wilt, 2024).

The etiopathogenesis of diabetes differs between the two main forms (I and II). Hence, in the first form, the main cause is represented by the autoimmune destruction of pancreatic beta cells, in people with a genetic predisposition, with the consequent decrease in insulin production. However, in type II diabetes, the pancreas produces insulin, but there are different degrees of insulin resistance precipitated by the complex interaction of genetic factors and environmental factors. In this situation insulin secretion is inadequate to meet the increased demand caused by insulin resistance (Calabria, 2022). The diagnosis of diabetes and prediabetes is similar to that of adults. For this purpose, fasting or random plasma glucose levels, at different times of the day, and/or glycosylated hemoglobin A1c levels are analyzed. Additionally, the presence or absence of characteristic symptoms (e.g., hyperglycemia, glycosuria, polydipsia, unexplained weight loss, nonspecific malaise) is taken into account (Calabria, 2022). Current guidelines note as targets a blood glucose level between 4 and 10 mmol/L (70–180 mg/dL). During the fasting period, the optimal range becomes narrower, of 4–8 mmol/L (70–144 mg/dL) (de Bock et al., 2022). Islet cell antibodies are not usually measured to diagnose type 1 diabetes. Being found in only about 5% of children, they are not considered to be specific markers (Los and Wilt, 2024). Except for the similarities in diagnosis with the adult form, pediatric diabetes must follow a separate management line. The possibility of overlapping other autoimmune diseases (e.g., thyroid damage, Addison’s disease, rheumatoid arthritis, systemic lupus erythematosus, psoriasis, inflammatory bowel diseases, autoimmune hepatitis, vitiligo) should be known in the case of children with type I diabetes (Calabria, 2022; American Diabetes Association Professional Practice Committee, 2022).

Baig S. et al. recently demonstrated that the hereditary character of diabetes can predispose to an increased sensitivity to inflammation and oxidative stress, thus accentuating the subsequent burden of the disease (Baig et al., 2020). Next, Wittenstein B. et al. support the existence of disturbances of the oxidative mechanisms since childhood (Wittenstein et al., 2002). This finding was also confirmed by Varvarovská J. et al., who demonstrated the existence of a tendency to overproduction of free radicals in the first degree relatives (siblings) of the patients (Varvarovská et al., 2003). In particular, Martín-Gallán P. et al. take as a model of debate the oxidative damage of lipids, proteins and DNA characteristic of diabetic patients with microangiopathy. They conclude that oxidative stress may represent an epicenter of the early development of diabetes-related complications (Martín-Gallán et al., 2007). In agreement with them, it has been shown that long-term exposure to high blood glucose concentrations can lead to increased inflammation and the oxidative component, with an impact on neuronal integrity, a neurotransmitters and kidney function. The findings are essential in order to optimize the treatment of pediatric diabetes. Although the authors emphasize the need for further investigations on the field of free radicals and oxidants-antioxidants, under-debated in pediatric populations (Kaya et al., 2015; Hernández-Marco et al., 2009; Arabshomali et al., 2023; Tuell et al., 2023; Çam et al., 2023).

Thus, the optimal monitoring of the organic antioxidant capacity is a key point in preventing the accelerated development of diabetic complications (Grabia et al., 2023). Further, Varvarovská J. et al. and Chiavaroli V. et al. underlines the importance of knowing and integrating the means of counteracting oxidative stress in the therapeutic scheme of children in order to minimize the degree of oxidative damage (Varvarovská et al., 2004; Chiavaroli et al., 2011). Natural antioxidant products (e.g., vitamin E, vitamin C, beta-carotene, selenium, manganese, polyphenols) may act to slow or prevent systemic decline. They act on several levels, among which we mention the reduction of mitochondrial oxidative stress, prevention of the harmful effects of lipid peroxidation and essential cofactors for antioxidant enzymes (Arabshomali et al., 2023; Tuell et al., 2023).

## 3 Place of oxidative stress in pediatric diabetes

Oxidative stress represents the disruption of the systemic balance between the production of oxidizing agents and the antioxidant defense. It has been incriminated over time both as a triggering factor and as a maintenance factor of diabetes and its comorbidities (Rains and Jain, 2011). In this sense, markers specific to oxidative stress were detected both in early and advanced disease. The pro-oxidative state can be induced by a disorganized lifestyle (inadequate diets, obesity, sleep restrictions, recurrent episodes of ketosis) or by the underlying condition (Kacarevic et al., 2020). Although reactive oxygen and nitrogen species are involved in numerous physiological processes, their excess causes damage to lipids, proteins, cell membranes or DNA (Tsukahara, 2007). The consequences of a pro-oxidative environment unfold in a double manner on the reference metabolic pathology. Thus, regarding the acute systemic damage, we can state that oxidative stress is involved in the production of pediatric diabetes, being correlated with the development of insulin resistance, β-cell dysfunction, impairment of glucose tolerance and mitochondrial dysfunction (Kacarevic et al., 2020). In the medium and long term, oxidative stress is implicated in the increase of associated comorbidities (e.g., nephropathy, neuropathy, retinopathy, cardiac or vascular damage). To illustrate, we take renal pathology as a reference point. One of the key factors in determining oxidative stress and accompanying renal dysfunction is NADPH oxidase 4 (NOX4). Added to this is nicotinamide phosphoribosyltransferase (NAMPT), regulator of the response to oxidative stress, apoptosis, lipid and carbohydrate metabolism, inflammation and insulin resistance. At the opposite pole, activation of the nuclear factor 2-erythroid-2 (NRF2) pathway has a strong antioxidant role (Wu H. et al., 2018; Garten et al., 2015).

**FIGURE 1:**
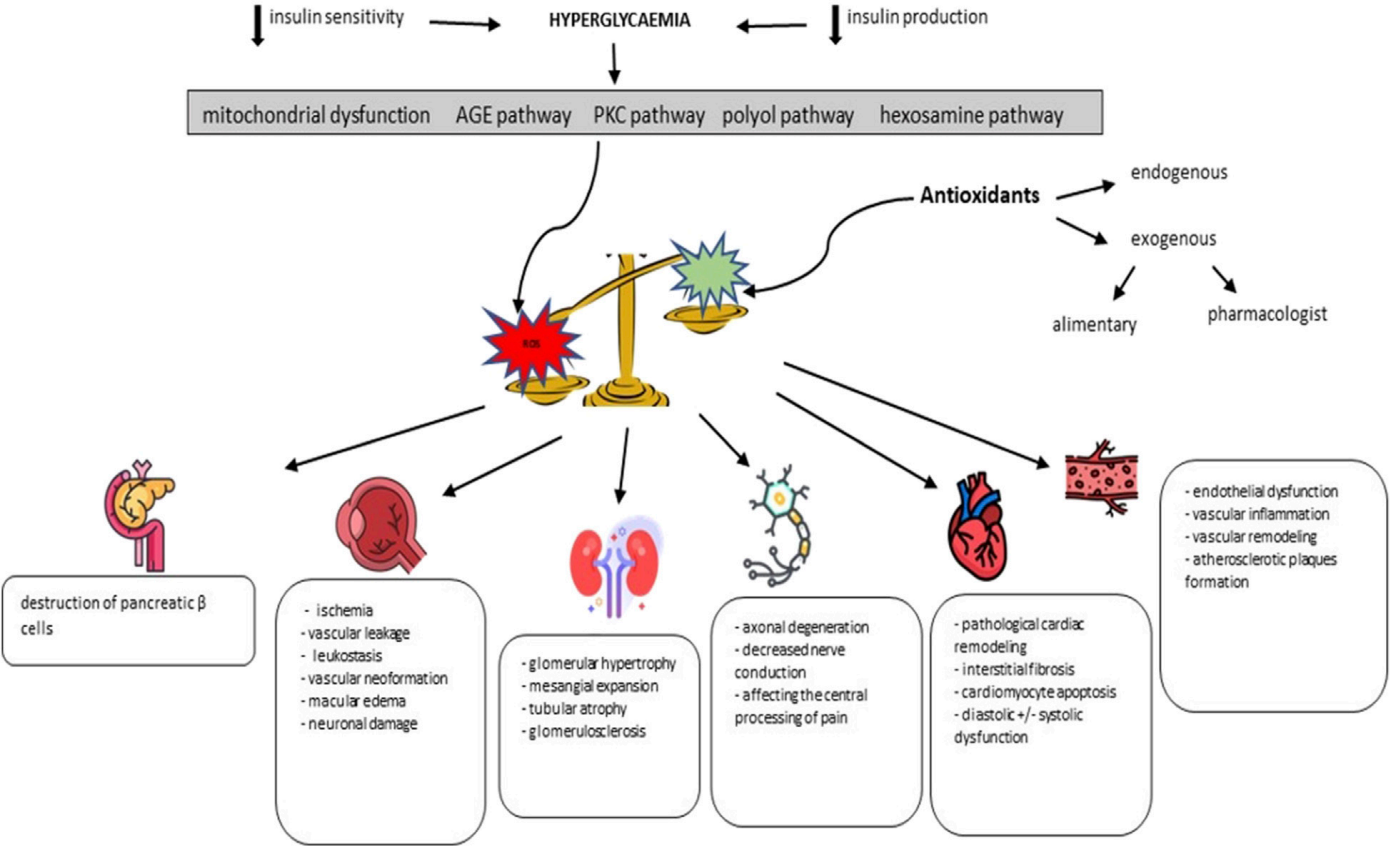
The role of oxidative stress in the pathogenic cascade of diabetic complications (adapted from Giacco F. et al. and Forbes JM. et al.) (Giacco and Brownlee, 2010; Forbes and Cooper, 2013).

Since the causal relationship regarding oxidative stress - pediatric diabetes is a complex one, we develop in the following the main findings currently available in the literature. We will thus review data regarding the involvement of oxidative stress in affecting pancreatic homeostasis, but also in the initiation and maintenance of retinopathy, nephropathy, neuropathy, cardiopathy and diabetic angiopathy. Summarizing the existing data in the literature, Figure 1 schematically exposes the pathogenic cascade identified in diabetes, with an emphasis on oxidative stress and its implications in the initiation and maintenance of chronic complications.

### 3.1 Destruction of pancreatic islets

Maintaining the structural and functional integrity of the β-pancreatic cells is a priority. Oxidative stress and inflammation have been shown to be disturbing factors of this balance (Saeedi Borujeni et al., 2019; Gorasia et al., 2015). The consequences are the disruption of immune tolerance following crosstalk between immune cells and β-cells. The main causes seem to be the alteration of signal transduction, enzyme activity, gene expression or ion channels, in parallel with antigen presentation and induction of apoptosis (Piganelli et al., 2021). Active or passive smoking represents an additional risk to those stated previously, synergistically with obesity. Nicotinamide has been shown to be beneficial in disease prophylaxis in high-risk individuals and stimulation of residual insulin secretion in newly diagnosed patients. Similarly, beneficial effects were also recorded in the case of N-acetylcysteine administration or the correlation of insulin dose with carbohydrate intake (Ludvigsson, 1993; Tong et al., 2020; Kostopoulou et al., 2020). At the same time, Lee J et al. posit that multiple cellular processes encountered in pancreatic dynamics are rhythmic and subject to circadian regulation (Lee et al., 2018).

Interleukin 6 (IL-6) has been intensively studied regarding its role in the specific oxidative dynamics of diabetes. It is certified that IL-6 represents the crossroads between autophagy and the antioxidant response. Although it is a pro-inflammatory cytokine, IL-6 seems to have a protective role in the pathogenesis of diabetes. The decrease in its signaling in β cells increases their susceptibility to oxidative damage. In parallel, IL-6 reduces reactive oxygen species and mitochondrial activity, stimulating mitophagy (Marasco et al., 2018). Recent studies confirm the correlation between the level of IL-6 and the complications of diabetes, both in murine models and among young patients (Karahmet et al., 2021; Robinson et al., 2020). Reis JS. et al. argue that the functional changes involved in the complications of diabetes may begin as early as the first years of diagnosis. Consequently, IL-6 is a key pawn in adaptation to stress, marking new research directions regarding targeted therapies (Rajendran et al., 2020; Reis et al., 2012).

### 3.2 Retinopathy

Retinopathy is classically defined as a complication of diabetes that structurally and functionally affects the posterior eye pole, with a frequency of approximately 15% since the time of diagnosis (Rodríguez et al., 2019). The physiopathological center of the disease is represented by ischemic tissue damage, later doubled by compensatory vascular neoformation (non-proliferative stage and proliferative stage) (Rivera et al., 2017). The mechanisms implicated in ocular tissue damage are atmospheric oxygen, hyperglycemia (through advanced glycosylation products, peroxisome proliferator-activated receptor γ disruption, epigenetic changes), inflammation, environmental chemicals, and free radical-catalyzed peroxidation of long-chain polyunsaturated acids. This results in toxic metabolites. Furthermore, oxidative stress is also involved in affecting the reendothelialization capacity (Guzman et al., 2017; Calderon et al., 2017; Gui et al., 2020). Wert KJ. et al. notes the involvement of superoxide dismutase-3 in vitro-retinal pathogenesis. This enzyme proved to be well represented in the vitreous body (Wert et al., 2018). Laser phototherapy, the administration of anti-vascular endothelial growth factor (VEGF) agents or vitrectomy are established therapeutic methods. Among the beneficial antioxidant substrates, we note the α lipoic acid, taurodeoxycholic acid, apocynin, polyphenols and vitamins (vitamin E, C, B1) (Rodríguez et al., 2019).

Oxidative stress can therefore precipitate the abnormalities induced by hyperglycemia and inflammation, mainly through the interaction with the polyol, hexosamine, angiotensin II pathways, the hyperactivation of protein kinase C isoforms (PKC) and the accumulation of advanced glycation end products (AGEs) (Wu MY. et al., 2018; Kang and Yang, 2020). In addition to these, epigenetic changes, DNA methylation and the abnormal activity of nuclear factors (e.g., strongly activated nuclear factor κB -NF-κB-), doubled by the attenuation of NRF2 activity, have been shown to be involved in the accumulation of free radicals (Duraisamy et al., 2018). All these changes form a vicious circle, leading over time to mitochondrial, microvascular dysfunction and apoptosis. Further, ischemia and local inflammation occur, which stimulate neovascularization, macular edema and neurodysfunction (Kowluru, 2023). In conclusion, oxidative stress represents an important pillar in the pathogenesis of diabetic retinopathy. In this sense, an individualized antioxidant therapy of patients can be obtained, centered on optical coherence tomography as a screening method. The technique seems to be able to evaluate the oxidative stress of the subretinal space. To this must be added the obtaining of an adequate glycemic control.

From a pharmacological point of view, the substances used in the management of diabetic retinopathy are diphenyleneiodonium, apocynin, triamcinolone acetonide, dexamethasone sodium phosphate, fluocinolone acetonide, statins, fenofibrate, or glucagon-like peptide 1 receptor agonists (GLP 1) (Rodríguez et al., 2019; Berkowitz, 2020). To these are added the previously mentioned antioxidant substances, doubled by microelements (zinc, copper, selenium, manganese), sirtuin pathway modulators (antagomiR, resveratrol, glycyrrhizin, melatonin), N-acetyl cysteine, benfotiamine, nicanartine, capsaicin, β-carotene, taurine, lutein, caffeic acid phenethyl ester, cannabidiol, 8-hydroxy-N, N-dipropyl-2-aminotetralin and green tea (Kang and Yang, 2020; Berkowitz, 2020; Li et al., 2017; Nebbioso et al., 2022; Liu et al., 2022; Tu et al., 2021).

### 3.3 Nephropathy

Diabetic nephropathy is another microvascular complication encountered in the evolution of diabetes with a frequency of up to 40% of cases (Darenskaya et al., 2023). The defining histological features are glomerular hypertrophy, basement membrane thickening, mesangial expansion, tubular atrophy, interstitial fibrosis, and arterial thickening (Darenskaya et al., 2023; Kashihara et al., 2010; Sagoo and Gnudi, 2018). Mamilly L. et al. underlines the positive correlation between the development of nephropathy and glycemic variability (Mamilly et al., 2021). However, the pathogenesis of renal damage in diabetic nephropathy is multiple and far from being completely elucidated. Among the newly introduced therapeutic targets in the field of research, we delimit oxidative stress which, together with hyperglycemia, forms a vicious cycle with a strong impact on metabolic homeostasis and organic functionality (Arora and Singh, 2014; Artenie et al., 2004). Several molecules compete in the modulation of oxidative stress, among which we note NADPH oxidase (subtypes 1, 2 and 4), xanthine oxidase, lipoxygenase, cytochrome P450, AGEs, disturbances of the polyol pathway, uncoupled nitric oxide synthase or the respiratory chain mitochondria. The goal is to reduce the risk of progression of diabetic nephropathy to chronic renal failure (Goycheva et al., 2023; Østergaard et al., 2020; Yamagishi and Matsui, 2010). It is known that chronic renal failure is a pathology accompanied by systemic dysbiosis, thus potentiating the occurrence of other diseases through the intestine-target organ axes. Among the pathologies whose list of precipitating factors includes dysbiosis, we mention inflammatory conditions, autoimmunity or atopy. At the same time, it is known about patients in the final stage of renal damage that they have a much-reduced capacity to compensate for the negative effects of oxidative stress (Pantazi et al., 2023; Mocanu et al., 2023; Lupu et al., 2023b; Lupu et al., 2024a; Lupu et al., 2024b; Lupu et al., 2023c; Lupu et al., 2023d; Ioniuc et al., 2024; Lupu et al., 2024c). The mechanism involved in renal decline is the increase in the expression of extracellular matrix genes following the stimulation of protein kinase C, mitogen-activated protein kinases and various cytokines and transcription factors by reactive oxygen species represented in excess. Added to this is the overexpression of the renin-angiotensin system (Kashihara et al., 2010; Sagoo and Gnudi, 2018).

The main organelles damaged by oxidative stress were found to be peroxisomes. Known for their multiple metabolic roles, they undergo functional changes depending on systemic conditions predisposing to the increase of free radicals. In this sense, studies on murine models have shown the benefit of supplementing with antioxidants in restoring peroxisomal balance and mitigating diabetic nephropathy (Dhaunsi and Bitar, 2004). Phytochemical substances (e.g., dietary antioxidants, Chinese medicinal plants, vitamins, trace elements - selenium, zinc -, catechins, coenzyme Q10, omega-3 fatty acids, resveratrol, curcumin, quercetin, soy, dioscin, α-lipoic or phenolic acid) are known for its beneficial effect on renal function and alleviation of oxidative stress (Hernandez et al., 2022; Gerardo Yanowsky-Escatell et al., 2020; Hu et al., 2022; Chen M. et al., 2023; Zhong et al., 2022). To these are added kelch-like ECH-associated protein 1, NRF2, lipoxin and glutathione peroxidase-1 representing antioxidant mechanisms whose integrity depends on the evolution of diabetic nephropathy (Darenskaya et al., 2023; Østergaard et al., 2020). Besides these, the functionality of metabolic pathways mediated by AMP-activated protein kinase (AMPK)/Sirtuin-1 (Sirt1), the transcription factor SIRT1-forkhead O (FOXO), Sirt1 and NF-κB can be influenced by oxidative stress (Jin et al., 2023). In addition, Peters V. et al. highlight the possible inclusion of carnosine (β-alanyl-L-histidine) among modern therapeutic approaches. The interest in this is partly due to its anti-inflammatory and antioxidant properties. The disadvantages of human administration reside in the reduced halving period (Peters et al., 2020).

Among the effective pharmacological therapies, we mainly discuss antidiabetics such as GLP1, dipeptidyl peptidase-4 (DPP-4) inhibitors, sodium-glucose transport protein 2 (SGLT2) and insulin. Adjuvants include Rapamycin, Ruboxistaurin, Pentoxifylline, the Ursolic Acid - Empagliflozin combination, aspirin and cyclooxygenase-2 (COX-2) inhibitors, 3-hydroxy-3-methylglutaryl CoA (HMG-CoA) reductase inhibitors, adiponectin and the monocyte chemoattractant protein inhibitor. 1 and 2 (MCP-1 and MCP-2) (Østergaard et al., 2020; Mima, 2013; Wu et al., 2021). To these is added the achievement of good glycemic control, the adequate maintenance of blood pressure and lipid profile, together with the approach of a healthy lifestyle (Singh et al., 2011).

### 3.4 Neuropathy

It manifests itself through intense pain and a decrease in the quality of life, reported in up to 1/3 of patients with diabetes, regardless of the associated neurological deficits (Pang et al., 2020). The physiopathological mechanisms directly involved are axonal degeneration occurring in cells with a reduced capacity to modulate their carbohydrate intake following chronic hyperglycemia and the excess production of reactive species (Ye et al., 2022). The indirect means that disrupt the neuronal balance are increased inflammation and mitochondrial dysfunction (Fernyhough and McGavock, 2014). In a secondary plan, we discuss the effect of genetic polymorphism in the pathogenesis of complications associated with diabetes (Babizhayev et al., 2015). It is also noted that pain processing in the central nervous system is compromised in diabetic neuropathy. Depending on the degree of damage and duration of symptoms, neuropathy can be divided into diffuse and focal (less frequent and often self-limited). The most common form is distal symmetrical polyneuropathy of the lower and upper extremities (Dewanjee et al., 2018; Sztanek et al., 2016).

There is an increased risk that patients with polyneuropathy will develop cognitive impairment (e.g., dementia). At the same time, maternal diabetes may predispose to fetal neurodevelopmental defects. Therefore, close follow-up of patients is essential. To facilitate this, Etienne I. et al. discuss the utility of correlating low malondialdehyde levels with the coexistence of diabetes mellitus and diabetic polyneuropathy (Etienne et al., 2019; Jin et al., 2016).

In combating oxidative stress and its effects on the quality of life of patients with diabetic neuropathy are essential substances such as α-lipoic acid, vitamins (A, C and E), acetyl L-carnitine, taurine, melatonin, quercetin and N–acetylcysteine (Pang et al., 2020; Zhang et al., 2021). Methylcobalamin is added to these, a substance that competes with α-lipoic acid in terms of therapeutic efficiency in reducing the sensation of numbness and paresthesia. In contrast, α-lipoic acid is recommended when the predominant complaints are burning and pain (Han et al., 2018). If in diabetic nephropathy Ruboxistaurin (specific inhibitor of protein kinase C) has proven therapeutic efficiency, in neuropathy aldose reductase inhibitors (Epalrestat, Ranirestat) and anti-AGE agents (Benfotiamine) have rather demonstrated a therapeutic effect. An effective solution appears to be the double association of phosphodiesterase inhibitors (Rolipram and/or Pentoxifylline), aiming both at reducing oxidative stress and the level of inflammatory factors (Dastgheib et al., 2022). For adjuvant purposes, it is possible to use antiepileptic drugs (Pregabalin, Gabapentin), antidepressants (Duloxetine), opioid analgesics (Tramadol) or non-steroidal anti-inflammatory drugs (Dewanjee et al., 2018). Dexmedetomidine (anxiolytic, analgesic and sedative derived from imidazole) and Hesperidin are also an approach considered promising in the future (Lin et al., 2023; Syed et al., 2023).

Future strategies focus on the use of hydrogen therapy in alleviating diabetic symptoms and reducing the morbidity caused by it. The benefits of its administration include reduction of blood glucose, improvement of motor nerve conduction velocity, mitigation of oxidative stress (reduction of malondialdehyde, reactive oxygen species and 8-hydroxy-2-deoxyguanosine), partial restoration of superoxide dismutase activities and regulation of NRF2 expression (Han et al., 2023).

### 3.5 Cardiovascular damage

The two entities that are the subject of this subchapter (diabetes and cardiocirculatory manifestations) have a common pathogenic background represented by low-grade chronic inflammation (Hariharan et al., 2022). Added to this is the impact of oxidative stress on cardiovascular and metabolic balance. Its main determinants appear to be obesity, diabetes, smoking and pollution (Hertiš Petek et al., 2022; Cojocaru et al., 2023; Niemann et al., 2017; Lupu A. et al., 2024). The role of intervention in modulating these variables is vital, its absence being accompanied by medium- and long-term consequences on the health of patients (for example, chronic inflammation in childhood increases the risk of atherosclerosis and cerebrovascular diseases). Also, there is evidence of the direct correlation between the level of oxidative stress, the relative risk of cardiovascular damage and mortality and the value of glycosylated hemoglobin (HbA1c), respectively the period of manifestation of diabetes. The link is objectified by the escalation of endothelial, inflammatory and pro-coagulant biomarker values among patients with poorly controlled diabetes (Hertiš Petek et al., 2022; Zhao et al., 2021; Domingueti et al., 2016; Seckin et al., 2006; Letunica et al., 2021). For an easier presentation of the subject, in what follows, we will subdivide the discussion into two directions of interest, namely, cardiac and vascular damage.

#### 3.5.1 Cardiomyopathy

The importance of the perinatal period (intrauterine and postnatal) in the subsequent optimal development of the child is well known. In this stage Topcuoglu S. et al. postulates the implications played by the incompletely compensated oxidative stress in determining the severity of the myocardial and hematological damage encountered in the first days of life of infants from diabetic mothers (Topcuoglu et al., 2015). Later, in evolution, diabetic cardiomyopathy and heart failure appear as consequences of metabolic and energetic disturbances induced by the absence/insensitivity to insulin associated with the underlying pathology. It is estimated that the risk increases by 30% - type 1 diabetes and respectively 8% - type 2 diabetes for each percentage increase in HbA1c (Jia et al., 2018). Therefore, there is an increase in oxidative stress, deregulation of calcium homeostasis, inadequate activation of the renin-angiotensin-aldosterone system, mitochondrial dysfunction, lipid peroxidation, post-translational modification of proteins and the activation of several inflammatory and signaling pathways involved in the promotion of cardiac oxidative stress that mediates cellular and extracellular lesions. Superoxide anion radicals produced in excess at the mitochondrial level induce the inhibition of glyceraldehyde 3-phosphate dehydrogenase, the increase in the flow of polyol and hexosamine pathways, the formation of excess AGEs, the activation of the receptor for AGEs (RAGE) and protein kinase C isoforms (Duncan, 2011; Chen et al., 2022; Peng et al., 2022; Tang et al., 2022; De Geest and Mishra, 2022). We encounter pathological cardiac remodeling, interstitial fibrosis and cardiomyocyte apoptosis, doubled by diastolic and systolic dysfunction. For an easier differentiation, Table 1 illustrates the main characteristics of cardiac damage depending on the form of diabetes manifested (Tan et al., 2020). In the occurrence of mitochondrial and cardiac changes induced by diabetes, a key role seems to be played by the regulation of signaling pathways targeting mitogen-activated protein kinases (e.g., extracellular signal-regulated kinase 1/2 -ERK1/2, n-terminal protein kinase c-Jun -JNK and p38 MAP kinase) (Xu et al., 2016).

**TABLE 1:** Characteristics of cardiac damage depending on the form of diabetes [adapted from Tan et al. (2020)].

Type of diabetes	Histological and physiopathological characteristics	Symptoms
Type 1 diabetes	• loss of cardiomyocytes • remodeling of the left ventricle • increased deposition of myocardial collagen • increased final diastolic volume in the left ventricle • systolic function disturbances	- heart failure with low ejection fraction
Type 2 diabetes	• coronary microvascular inflammation • paracrine-mediated damage to cardiomyocytes and endothelial cells • concentric left ventricular remodeling and hypertrophy • increased rigidity • diastolic dysfunction	- heart failure with preserved ejection fraction, minimally symptomatic (assessed by tissue Doppler imaging) - late in the evolution of heart failure with low ejection fraction

The three main directions in the fight against the excessive production of oxygen radicals induced by hyperglycemia, hyperlipidemia and hyperinsulinemia/increased insulin resistance are endogenous antioxidant enzymes (SOD, catalase, glutathione peroxidase, NRF2, heme oxygenase (HO)-1, redoxins, aldehyde oxidases or sirtuins), the administration of targeted pharmacological agents (mito-TEMPO, Resveratrol, Piceatannol, Quercetin, Taxifolin, antidiabetics, angiotensin-converting enzyme inhibitors, angiotensin receptor antagonists, renin inhibitors) and respectively increased exogenous intake of substances with antioxidant effect (Byrne et al., 2021; Huang et al., 2020). Among the food components with cardioprotective effects we mention flavonoids (e.g., Naringenin), sulforaphane, N-acetyl cysteine, zinc (metallothionein cofactor), selenium, vitamins E and C, β-carotene, α-lipoic acid, omega 3, coenzyme Q (Tan et al., 2020; Byrne et al., 2021; Thandavarayan et al., 2011; Xu et al., 2023). In murine models, Zhang C. et al. demonstrated the benefit of non-mitogenic acid fibroblast growth factor administration over a period of 6 months in the prophylaxis of diabetic cardiomyopathy (Zhang et al., 2013). Other substances that have demonstrated benefits are ursolic acid, curcumin, galangin, acacetin, fisetin, dihydromyricetin or isoliquiritigenin (Wang et al., 2018; Ren et al., 2020; Abukhalil et al., 2021; Song et al., 2022; Althunibat et al., 2019; Chen Y. et al., 2023; Gu et al., 2020).

#### 3.5.2 Angiopathy

The systemic pro-oxidative status characteristic of diabetes brings about changes in the integrity of the vascular network from the early stages. By following a group of 35 patients with type 1 diabetes, Suys B. et al. hypothesize that the circulating level of copper/zinc SOD (also known as SOD3) can be correlated with vascular damage and the degree of arterial dilatation mediated by the flow (Suys et al., 2007; Su et al., 2008). Further, El Samahy MH. et al. note that the carotid intimate-medium thickness correlates positively with nitric oxide values and negatively with systemic antioxidant capacity (El et al., 2013). Ahmad FA. et al. widened the horizons of screening by demonstrating the correlation between epicardial fat thickness (limit value for prediction: 6.95 mm) measured echocardiographically and vascular damage (Ahmad FA. et al., 2022). Studies on murine models also facilitated the inclusion of microRNA (miRNA), respectively miR-30d/e, among possible relevant biomarkers in determining the degree of coronary microvascular damage. At the same time, it represents a useful prophylactic measure in preventing compromise of diastolic function and progression to heart failure with preserved ejection fraction (Veitch et al., 2022). In conclusion, Koutroumani N. et al. note the existence among children with diabetes of a protective mechanism against oxidative stress and angiopathy centered on the different modulation of the AGE receptor (Koutroumani et al., 2013).

Regarding the exogenous means of antioxidant protection, among them we find vitamins (A, C, E, complex B), polyphenols, glutathione, α-lipoic acid, carotenoids, trace elements (copper, zinc and selenium), coenzyme Q, taurine, tetrahydrobiopterin, L-arginine, folic acid or acetylcysteine. Effective pharmacological therapy is represented by 3-hydroxy-3-methylglutaryl coenzyme A reductase inhibitors (statins), although they do not show as a mechanism of action the reduction of oxidative stress. Fibrates, angiotensin-converting enzyme inhibitors, metformin and fish oils can be added to this (in combination or not) (Laight et al., 2000; Suganya et al., 2016; Yim et al., 2007; Hamilton et al., 2007). For the pediatric population, the judicious use of cocoa and dark chocolate can be considered an attractive option in order to increase cardiovascular protection from the perspective of their antioxidant capacities (Grassi et al., 2013).

Undoubtedly, the damage to the myocardial vasculature benefits from increased attention in part because of the cardiac consequences resulting from its disruption. It should not be neglected, however, that angiopathy due to oxidative/nitrogen stress is a diabetic complication that affects all the target organs (eyes, kidneys, nervous system), disrupting the homeostatic balance and thereby precipitating related comorbidities (Santilli et al., 2004; Tsukahara et al., 2003; Grochowski et al., 2018). Further, Table 2 presents a synthesis of the main therapeutic means (pharmacological and non-pharmacological) that can be successfully approached in the curative and adjuvant management of diabetes complications.

**TABLE 2:** Targeted therapeutic targets in the prophylactic/curative management of diabetic comorbidities.

Affect	Endogenous antioxidants	Exogenous antioxidants	Pharmacological/alternative substances^a^
Retinopathy	SOD	α lipoic acid	Antidiabetics/Insulin
	CAT	Taurodeoxycholic acid	Anti-VEGF
	TrxR	Apocynin	Vitrectomy
	GR	Taurine	Diphenyleneiodonium
	GPX	Lutein	Apocynin
	GSH	Phenethyl ester of caffeic acid	Triamcinolone acetonide
		polyphenols	Dexamethasone sodium phosphate
		Vitamins (E, C, B1)	Fluocinolone acetonide
		Trace elements (zinc, copper, selenium, manganese)	Statins
		Sirtuin pathway modulators (AntagomiR, Resveratrol, Glycyrrhizin, Melatonin)	Fenofibrate
		N-acetyl cysteine	
		Benfotiamine	
		Nicarnitine	
		Capsaicin	
		β-carotene	
		Cannabidiol	
		Green tea	
Nephropathy	kelch-like ECH-associated protein 1	Chinese medicinal plants	Antidiabetics/Insulin
	NRF2	Soy	Rapamycin
	Lx	Vitamins	Ruboxistaurin
	GPx-1	Trace elements (selenium, zinc)	Pentoxifylline
		Catechins	Ursolic acid – Empagliflozin
		Coenzyme Q10	Aspirin and COX-2 inhibitors
		Omega-3 fatty acids	HMG-CoA reductase inhibitors
		Resveratrol	Adiponectin
		Curcumin	MCP-1 and MCP-2
		Quercetin	
		Dioscine	
		α-lipoic acid	
		Phenolic acid	
Neuropathy		α-lipoic acid	Antidiabetics/Insulin
		Vitamins (A, C, E)	Aldose reductase inhibitors (Epalrestat, Ranirestat)
		Acetyl L-carnitine	Anti-AGE agents (Benfotiamine)
		Taurine	Phosphodiesterase inhibitors (Rolipram and/or Pentoxifylline)
		Melatonin	Antiepileptics (Pregabalin, Gabapentin)
		Quercetin	Antidepressants (Duloxetine)
		N-acetylcysteine	Opioid pain relievers (Tramadol)
		Methylcobalamin	NSAIDs
Cardiomyopathy	SOD	Flavonoids	Antidiabetics/Insulin
	CAT	Sulforaphane	mito-TEMPO
	GPX	N-acetyl cysteine	Resveratrol
	NRF2	Trace elements (zinc, selenium)	Piceatannol
	HO-1 redoxins	Vitamins (E, C)	Quercetin
	aldehyde oxidase	β-carotene	Taxifolin
		α-lipoic acid	ACE
		Omega 3	ARBs
		Coenzyme Q	renin inhibitors
		Ursolic acid	
		Curcumin	
		Galangin	
		Acacetin	
		Fisetin	
		Dihydromyricetin	
		Isoliquiritigenin	
Angiopathy	SOD	Vitamins (A, C, E, B complex)	statins
		trace elements (copper, zinc, selenium)	fibrates
		Polyphenols	ACE
		Glutathione	Metformin
		α-lipoic acid	
		Carotenoids	
		Coenzyme Q	
		Taurine	
		Tetrahydrobiopterin	
		L-arginine	
		Folic acid	
		Acetylcysteine	

SOD, superoxide dismutase; CAT, catalase; TrxR, thioredoxin reductase; GR, glutathione reductase; GPX, glutathione peroxidase; GSH, reduced glutathione; NRF2, nuclear factor erythroid 2–related factor 2; Lx, lipoxin; GPx-1, glutathione peroxidase −1; HO-1, heme oxygenase (HO)-1; VEGF, vascular endothelial growth factor; MCP-1 and MCP-2, monocyte-chemoattractant protein inhibitor-1 and 2; HMG-CoA, reductase, 3-hydroxy-3-methylglutaryl CoA reductase; COX2, cyclooxygenase-2; ARBs, angiotensin receptor antagonists; ACE inhibitors, angiotensin-converting enzyme inhibitors; NSAIDs, nonsteroidal anti-inflammatory drugs.

^a^ Prescription conditions and dose adaptation according to age/comorbidities are done in accordance with international guidelines and recommendations.

## 4 The main antioxidant substances used in management

We exposed in the initial part of the article the role played by the imbalance between the formation and clearance of free radicals, substances that give the internal environment a pro-oxidant character. Its effects are felt both in the initiation, as well as in the maintenance and aggravation of diabetic manifestations. It is proven that the neutralization of reactive molecules can significantly decrease the risk of developing endothelial dysfunction, atherosclerosis, cardiomyopathy, retinopathy, nephropathy and neuropathy. However, the current medical literature rather encourages the use of antioxidants as a prophylactic means to prevent the accumulation of reactive free radicals, rather than for the purpose of cleaning them (Zatalia and Sanusi, 2013; Johansen et al., 2005). Focusing on the category of food antioxidants, with possible parallels in the pharmaceutical industry, we continue to develop the basic characteristics of the most popular substances and how they interfere with the prophylactic and curative management of diabetes and its complications. In addition, we will refer to foods with high antioxidant content, with the aim of popularizing a nutritious, balanced diet.

### 4.1 Vitamins (A, C, E and B complex)

Vitamin A represents a group of fat-soluble retinoids, with implications on growth, differentiation and cell signaling (through the intranuclear receptor), gene regulation, muscle integrity, resistance to infections and immunological balance. The optimal dose is between 200 and 500 μg/day (Martini et al., 2020; Iqbal and Naseem, 2015). Vitamin A is found in increased quantities in the following foods: liver and liver products, kidney and offal, oily fish and fish liver oils, eggs, carrots, red peppers, spinach, broccoli and tomatoes (Webster-Gandy et al., 2006).

Vitamin C (ascorbic acid) is a water-soluble vitamin with a role in the integrity of the connective tissue, the modulation of systemic enzymatic reactions and the functions of the central nervous system. At the same time, vitamin C increases nitric oxide production in endothelial cells by stabilizing the nitric oxide synthase cofactor, thereby neutralizing reactive oxygen species. Appropriate daily doses are between 35 and 75 mg (Johansen et al., 2005; Martini et al., 2020). Foods rich in vitamin C found in the daily diet are kiwi fruit, citrus fruit (oranges, lemons, satsumas, clementines, etc.), black currants, guava, mango, papaya, pepper, brussels sprouts, broccoli and sweet potato (Webster-Gandy et al., 2006).

Vitamin E is a fat-soluble vitamin (along with vitamin A), subdivided into 8 isoforms, of which the most active and intensively studied in the human species is α-tocopherol. Tocopherol reacts with the hydroxyl radical, neutralizing it by forming a stabilized phenolic radical that will later be reduced to the phenolic ring (Zatalia and Sanusi, 2013). Giannini C. et al. demonstrate through the cross-over, double-blind study, over a period of 12 months, the benefit brought by supplementation with high doses of vitamin E (1,200 mg/day) on the reduction of pro-oxidative markers and the improvement of antioxidant defense. However, supplementation could not restore the damage already done (Giannini et al., 2007). Later, Dotan Y. et al. disapprove the supplementation without discrimination, basing his statement on the results of recent meta-analyses that validate its correlation with increased mortality. The authors emphasize the need to outline some indications for its use in order to reduce the consequences, among them are the people known to be subject to oxidative stress (Dotan et al., 2009). Among the food’s rich in vitamin E, recommended for daily consumption, we note wheat germ oil, almonds, sunflower seeds and oil, safflower oil, hazelnuts, peanuts, peanut butter and corn oil (Webster-Gandy et al., 2006).

The B vitamin complex is made up of thiamin (B1), riboflavin (vitamin B2), niacin (B3), pantothenic acid (B5), pyridoxine (B6), biotin (B7), folic acid (B9) and cobalamin (B12). These vitamins are water-soluble and target various physiopathogenic stages during oxidative stress (Martini et al., 2020; Webster-Gandy et al., 2006). B1 plays an essential role in the oxidative decarboxylation of multienzyme branched chain ketoacid dehydrogenase complexes of the citric acid cycle. At the same time, its level can mark the neuroprotection/neurodegeneration balance (Depeint et al., 2006; Gibson and Zhang, 2002). B2 is a necessary cofactor for the flavoenzymes of the respiratory chain, glutathione reductase, simultaneously mitigating oxidative reperfusion injuries and lipid peroxidation (Depeint et al., 2006; Ashoori and Saedisomeolia, 2014). In parallel, B3 is a cofactor in the synthesis of NADH, being important in the economy of the amount of protons required for oxidative phosphorylation (Depeint et al., 2006). B5 is a necessary substrate for the formation of coenzyme A. It is also involved in the dynamics of the alpha-ketoglutarate and pyruvate dehydrogenase complexes, but also in the oxidation of fatty acids (Depeint et al., 2006). B6 is mentioned as an enzyme cofactor in more than 140 chemical reactions. Distinct from this, B6 has properties of reactive oxygen species scavenger, being a strong antioxidant, metal chelator and carbonyl cleaner (Depeint et al., 2006; Mooney and Hellmann, 2010; Wondrak and Jacobson, 2012). B7 is required for gluconeogenesis and fatty acid oxidation (Iqbal and Naseem, 2015). B9, together with its metabolite (5-methyltetrahydrofolate) regulates the bioavailability of nitric oxide by increasing the production and coupling of endothelial nitric oxide synthase. Thus, it interferes directly with the cleaning of superoxide radicals (Stanhewicz and Kenney, 2017). Mainly, B12 has the role of stabilizing DNA, being a cofactor for enzymes such as methionine synthase and methylmalonyl-CoA mutase. At the same time, it is involved in cleaning free radicals and reducing oxidative stress (Depeint et al., 2006; Halczuk et al., 2023).

In conclusion, the B vitamin complex is involved in ameliorating the toxicity induced by oxidative stress. However, supplementation must be judicious, Kandel R. et al. noting that prolonged exposure to large amounts of folic acid can induce acute renal cytotoxicity and fibrotic changes. The risk was reduced by the addition of N-acetylcysteine. Therefore, the use of vitamins from the B complex must be judicious. Of these, we note as a dosage standard a requirement of 0.7–2 μg/day of vitamin B12, respectively 110–320 μg/day of folic acid (Kandel and Singh, 2022). Food sources rich in these are eggs, milk and milk products (e.g., cheese, yoghurt), liver, kidney, yeast extracts, fortified breakfast cereals, bananas, yeast, offal, peanuts, nuts, brussels sprouts, cabbage, kale, spinach, broccoli, cauliflower, chickpeas, green beans, icebergs, lettuce, beans, peas, spring greens, potatoes, brown rice, whole grain pasta, beef, pork, chicken, salmon, game, wheat flour and maize flour (Webster-Gandy et al., 2006).

### 4.2 Minerals (zinc, copper, selenium, magnesium)

Their main role in the systemic oxidative economy is attributed to their function as enzyme cofactors. Taking zinc as an example, it modulates metallothionein activity. Protein rich in cysteine, metallothionein has the property of binding metals, modulating cellular and immune homeostasis. Thus, it exerts its antioxidant, antiapoptotic, detoxifying and anti-inflammatory effects. Intervenes in the signaling pathways induced by stress, thereby imprinting the evolutionary course of diabetes (Park et al., 2018). Foods recommended in the daily diet to avoid microelement deficiencies are lamb, green vegetables, pulses, leafy and root vegetables, crabs and shellfish, beef, offal, whole grains, pork, fish, poultry, milk and milk products, eggs, nuts (Webster-Gandy et al., 2006).

### 4.3 Glutathione

It represents one of the major redox buffering systems. Its roles are bidirectional, acting both as a direct scavenger and as a cofactor for the enzyme glutathione peroxidase (Johansen et al., 2005). In order to supplement it, the current literature notes that the oral intake of preformed glutathione, the supplementation of other constituents that regulate its level (e.g., N-acetylcysteine, omega 3, riboflavin, vitamin C, vitamin E, α-lipoic acid, selenium) are also possible options. and the increased intake of protein foods, in parallel with the optimal maintenance of gastric hydrochloric acid and pancreatic enzymes necessary for digestion. Food sources rich in glutathione are asparagus, broccoli, green beans, lemon, grapefruit, mango, avocado, banana, orange, papaya, parsley, potato, red pepper, bell pepper, spinach, strawberry, tomato, cucumber, carrot, yellow squash, cauliflower (Minich and Brown, 2019).

### 4.4 Polyphenols

These are compounds found mainly in foods of vegetable origin, which have antioxidants, anti-inflammatory and immunomodulatory properties. It exerts its antidiabetic role mainly by reducing the intestinal absorption of glucose, increasing pancreatic insulin secretion, modulating the intestinal microbiota and its metabolites. Depending on the chemical structure, polyphenols are divided into four groups, respectively flavonoids, stilbenes, phenolic acids and lignans. The number of phenolic units and their combination leads to different physical, chemical and biological properties. A representative model is perhaps the antithesis of flavonoids (e.g., Quercetin) - stilbenes (e.g., Resveratrol, Curcumin) (Jin et al., 2023). The main sources of dietary flavonoids are pomelo, blueberries, roselles, oranges, grapefruit, lemons and limes (Sapian et al., 2021).

Quercetin is a bioflavonoid compound (flavonol), composed of 15 carbon atoms joined in the form of two benzene rings and a heterocycle. It has demonstrated effects in the suppression of cell proliferation, inflammation and oxidative stress, in part by regulating glycolipid metabolism and improving the bioavailability of nitric oxide (Zhang et al., 2021; Sapian et al., 2021). At the molecular level, it activates the NRF2/HO-1 pathway, suppresses the AGE-RAGE pathway, improves the expression of SOD, GPx and CAT, increases the content of high-density lipoproteins, simultaneously reducing the expression of triglycerides, low-density lipoproteins and the expression of inflammatory molecules (Jin et al., 2023). Renal, one of the mechanisms of action, according to Wan H. et al., is the miR-485-5p/yes-associated protein (YAP1) pathway. YAP1 is a multipotent protein, involved in various processes such as osteoblast differentiation, tumor biology and diabetes complications (e.g., it promotes kidney damage). Its modulation favors the improvement of interstitial fibrogenesis (Wan et al., 2022). Its effects are not limited to the nephrotic component, the consumption of flavonol stopping retinal degeneration, offering cardio and neuroprotection by controlling apoptosis, inflammation, neurodegeneration and cardiac remodeling that occur following oxidative stress. In conclusion, it is noted that the optimal intake of quercetin (90–150 mg/kg/day, for 2–4 months) improves renal function, increases the formation of nephrin and podocin, decreases desmin, improves the deletion of podocytes and improves renal histology. Foods rich in quercetin are onions, cabbage, lettuce, tomatoes, grapes, apples and berries (Jubaidi et al., 2021; Ola et al., 2018).

Resveratrol has a similar action to quercetin, but a different chemical structure, resveratrol activates antioxidant enzymes and decreases the secretion of superoxide anion, hydroxyl radical, and inflammatory cytokines by modulating the NRF2/kelch-like ECH-associated protein 1 signaling pathway and AMPK expression. Additionally, it modulates Cyto C transport activity, thus delaying the progression of podocyte and tubular apoptosis (Jin et al., 2023). Curcumin is an extract of medicinal plants with significant antioxidant properties. It can reduce hypoxia and prevent angiogenesis by modulating hypoxia-inducible factor 1. It is noted that curcumin supplementation can inhibit VEGF expression (Li et al., 2017).

### 4.5 Coenzyme Q10

This is an endogenous, fat-soluble compound, considered to act on the electron balance of complex II in the mitochondrial transport chain. It is noted that, in high concentrations, it helps the clearance of free radicals, thereby improving endothelial function (Johansen et al., 2005). Food sources of coenzyme Q10 are mainly meat, fish, nuts and some oils. To these, dairy products, vegetables, fruits and cereals are added, in a significantly lower quantity. It is also worth mentioning the variability of the concentration of coenzyme Q10 in foods depending on the geographical area from which they come (Pravst et al., 2010).

### 4.6 Melatonin (N-acetyl-5-methoxy tryptamine)

It is considered one of the miracle molecules at the moment, performing numerous roles such as cleaning free radical species, regulating insulin metabolism, supporting the immune system, slowing down aging, counteracting insomnia, systemic inflammation, malignancies, periodontal pathologies, neuroprotection, mood balancing, sexual maturation and body temperature control. Looking specifically at the role of melatonin in counteracting systemic oxidative stress, we know that it plays the role of scavenger of excessive free radicals, more potent compared to nicotinamide adenine dinucleotide phosphate (NADPH), vitamin C and vitamin E. It also increases the efficiency of the electron transport chain in the mitochondria, thereby reducing the generation of free radicals and preventing the leakage of electrons. Last but not least, melatonin interferes in a beneficial sense with the generation and potentiation of endogenous antioxidants (e.g., glutathione, SOD, CAT, GPx, nitric oxide synthase) (Zephy and Ahmad, 2015; Nishida, 2005). Food sources of animal origin are meat (lamb, beef, pork, fish, chicken), eggs and dairy products. Vegetable sources (e.g., corn, rice, wheat, barley, oats, strawberries, cherries, grapes, walnuts, pistachios, almonds, tomatoes, peppers, mushrooms, black/white mustard seeds, soybeans) are subject to variability in the amount of melatonin dictated by the environment in which they are grown (e.g., temperature, duration of exposure to sunlight, ripening process, agrochemical treatment) (Meng et al., 2017).

### 4.7 α-lipoic acid

It is included in the category of hydrophilic antioxidants, exerting its effects in both aqueous and lipid environments. By reducing it to dihydrolipoate, dietary α-lipoic acid participates in the regeneration of other antioxidants (e.g., vitamins C and E, reduced glutathione), thus being considered an inducer molecule (Johansen et al., 2005). The endogenous source is a cofactor for mitochondrial α-keto acid dehydrogenases, while exogenous sources cannot be used in this sense. However, the exogenous supply of α-lipoic acid stimulates a series of biochemical reactions with pharmacotherapeutic value. There are added interactions with systemic inflammatory status, redox balance, the NRF2/kelch-like ECH-associated protein 1 pathway, glycolipid metabolism or metal chelation. α-lipoic acid is therefore considered a strong antioxidant, with insulin-mimetic and anti-inflammatory activity (Shay et al., 2009; Rochette et al., 2015; Packer et al., 1995). Food sources are muscle meat, heart, kidney and liver, and to a lesser extent fruits and vegetables. They cannot supply the entire organic requirement, thus recommending the use of food supplements as the primary source of nutrients (Shay et al., 2009).

### 4.8 N-acetyl cysteine

It is a vegetable antioxidant, precursor of glutathione, usually found in onions. Its role in oxidative dynamics is bivalent, in accordance with other molecular agents of the “thiol” type. N-acetyl cysteine is therefore involved both in cleaning reactive oxygen species and hydroxyl radicals (antioxidant effect), as well as in reducing transitional metals (pro-oxidative effect) (Šalamon et al., 2019).

In conclusion, we consider it appropriate to discuss the results obtained by Neri S. et al. regarding the importance of combining antioxidants (in this case N-acetylcysteine, vitamin E and vitamin C) in counteracting oxidative stress and preventing the harmful effects of a disorganized lifestyle (Neri et al., 2005). Similar results were observed in studies on adults and regarding associations such as vitamin A-vitamin E-zinc or high doses of B complex vitamins. These combinations led to the improvement of glycemic control, β-cell function and insulin secretion, while also promoting myelination, cellular metabolism and energy storage (Ford et al., 2018; Said et al., 2020).

## 5 Practical implications of the use of antioxidants in pediatrics

Pediatric patients with diabetes face several distinct challenges that include both physiological, psychological and developmental aspects. Among the most important in this regard, we mention incomplete metabolic development and the vulnerability of organs to glycemic fluctuations, which can cause damage to target organs (e.g., heart, kidneys) from an early age. At the same time, children face difficulties in maintaining a stable glycemic control due to growth, hormonal fluctuations and constant changes in physical activity. Oxidative stress is reported both in times of hyperglycemia and hypoglycemia. Last but not least, the immaturity of neural connections predisposes children’s brain tissue to hypersensitivity in response to systemic oxidative stress. Over time, this produces cognitive and emotional disorders, which can escalate to social maladjustment and adherence to treatment in the morning through denial/unawareness of the disease. Antioxidant interventions and the reduction of oxidative stress through drugs or specific supplements can contribute to reducing inflammation, protecting organs from the negative effects of free radicals and better stabilizing blood sugar levels. Ultimately, these interventions can increase the quality of life of children with diabetes, reducing the risk of complications and supporting healthy development (Brownlee, 2005; Bacha and Klinepeter Bartz, 2016; Matough et al., 2012; Silverstein et al., 2005).

Regarding the antioxidants mentioned above, there are clinical trials in the literature that debate the effectiveness of their use in pediatric diabetes depending on the choice of the moment of intervention. Thus, we note the research carried out by Ludvigsson J. et al. in the form of a randomized, double-blind study, carried out over 3 years and which included 46 children with type 1 diabetes at onset. The active substances used in comparison with placebo were nicotinamide, vitamin C, vitamin E, beta-carotene and selenium. The doses were adapted according to body weight. The monitored parameters were insulin requirement, C peptide, blood glucose and HbA1c. The statistical analysis of the data did not objectify a significant difference in the dynamics of the two groups. Consequently, the authors claim that the initiation of supplementation with high doses of antioxidant agents at the diagnosis of diabetes does not demonstrate the effectiveness of preserving beta cell function or metabolic balance. However, we do not lose sight of the absence of negative side effects, an aspect that requires the continuation of studies in various stages of the pathology (Ludvigsson et al., 2001).

Further research was undertaken with particular reference to substances such as vitamin D, the B vitamin complex, zinc or magnesium. About the first one, Giri D. et al. notes through the retrospective analysis of a cohort of 271 children with type 1 diabetes that the supplementation of 25-hydroxy vitamin D supplements led to the improvement of HbA1c values. The effectiveness of the intervention was dependent on the pre-treatment values of HbA1c and 25 hydroxy vitamin D (Giri et al., 2017). Similarly, a randomized double-blind study undertaken by Sharma S. et al. attested to the increased prevalence of vitamin D deficiency among children with type 1 diabetes. The intervention consisted of oral supplementation, monthly, for a period of 6 months, with doses of vitamin D between 60,000 IU and 120,000 IU (depending on age child). It was thus observed that, in the group that benefited from the intervention in addition to standard insulin therapy, C peptide levels were significantly higher, without a difference in terms of HbA1c and insulin requirements. The authors therefore conclude that oral vitamin D can be used in the adjuvant treatment of type 1 diabetes with the aim of increasing the residual function of beta cells and improving insulin secretion (Sharma et al., 2017).

Next, regarding the complex of B vitamins (B1, B6, B12), Elbarbary NS. et al. followed in a randomized controlled manner, the effects of oral supplementation on the dynamics of pediatric diabetic nephropathy. The evaluation was carried out over a period of 12 weeks, the monitored indicators being plasma homocysteine, HbA1c, urinary albumin excretion (EAU) and cystatin C. The statistical analysis demonstrates the effectiveness of the intervention on improving glycemic control and renal function, doubled by the objectification of a negative correlation between the initial value of cystatin C and the level of vitamin B12 (Elbarbary et al., 2020).

Finally, the implications of supplementing with trace elements are a controversial subject. So, Lobene AJ. et col. evaluated the effectiveness of zinc supplementation (9 mg of elemental zinc) over a period of 4 weeks, among healthy girls aged between 9 and 11 years. Statistical data were equivocal, indicating that the chosen supplementation method (dose and duration) was not optimal for influencing insulin secretion or insulin resistance in healthy adolescent females (Lobene et al., 2017). Subsequently, Matter RM. et al. analyzed in particular the population with diabetes in the context of β-thalassemia major. In this situation, oral supplementation with zinc 40 mg/day for 12 weeks demonstrated effectiveness in reducing iron load, lowering glycemic levels, increasing insulin secretion and improving glycemic control, without notable adverse effects (Matter et al., 2020). There were similar results in the case of magnesium supplementation among children with diabetes and associated hypomagnesemia. Shahbah D. et al. demonstrated positive correlation of magnesium with high-density lipoprotein, mean body volume and platelet count and negative with age, HbA1c, triglycerides, total cholesterol, low-density lipoprotein and duration of diabetes. Supplementation with 300 mg of magnesium oxide for 3 months demonstrated the effectiveness of improving glycemic control and the lipid profile (reduction of the atherogenic lipid fraction, doubled by the increase of the protective lipid fraction) (Shahbah et al., 2017).

Although the implications of the manipulation of the oxidative status in reducing the damage caused by diabetes in children are in full research, a bias identified in the reporting resides in the poor certification of the effect of specific antioxidants in the evolution of complications. This may be due in part to a long period of follow-up of human patients needed to establish such correlations. The data obtained from murine, *in vivo* or vitro studies being more generous. Consequently, we encourage the concentration of global efforts on obtaining data on the effectiveness, administration regimen and safety of the use of antioxidants in the chronic management of diabetic complications. Thus, an important aspect of the supplement is represented by the knowledge of the bioavailability of the substances involved. Table 3 summarizes the most important observations regarding exogenous antioxidants possibly involved in the evolutionary dynamics of pediatric diabetes.

**TABLE 3:** Bioavailability of the main exogenous antioxidants (Webster-Gandy et al., 2006; Carazo et al., 2021; Maurya et al., 2020; Subroto et al., 2021; Borel et al., 2013; Hrubša et al., 2022; Lindschinger et al., 2019; Maares and Haase, 2020; Ferreira et al., 2021; Pardo et al., 2021; Kaşıkcı and Bağdatlıoğlu, 2016; Kemper et al., 2022).

Class	Substance	Doses recommended in pediatrics	Remarks
Vitamins	Vitamin A	0–1: 350 ug/day 1–3: 400 ug/day 4–10: 500 ug/day 11–14: 600 ug/day Males 15+: 700 ug/day Females 15+: 800 ug/day Toxicity: more than 100 mg Toxicity signs: - Acute: vomiting, abdominal pain, anorexia, blurred vision, headache, and irritability - Chronic: headache - muscle and bone pain, ataxia, skin disorders, alopecia, liver toxicity, and - hyperlipidaemia. *Not all the chronic symptoms are reversible*.	Sources: diet, oral, intramuscular or topical medications Absorption: predominantly intestinal in diet/oral forms of administration Conversion rate dependent on: dose, body retinol levels and dietary fat content Circulation of retinoids: bound to proteins Excretion: renal or hepatic (bile) Medications (e.g., estrogens and oral contraceptives) stimulate retinoid absorption, while alcohol inhibits vitamin A metabolism Vitamin A susceptible to oxidation of its dense structured electrical ridges, for bioavailability various encapsulation techniques are used
	Vitamin C	0–1: 25 mg/day 1–10: 30 mg/day 11–14: 35 mg/day Males 15+: 40 mg/day Females 15+: 40 mg/day Toxicity: 1–10 g/day Toxicity signs: - diarrhoea and risk of kidney oxalate stone formation - sudden cessation of high dose supplements may precipitate rebound scurvy	Absorption: gastrointestinal tract by active transport and passive diffusion The rate of gastric emptying may affect absorption, so co-administration with food or use of slow-release forms may improve absorption Factors influencing bioavailability: pH (acidity of the environment affects absorption of vitamin C), interaction with other compounds (e.g., organic compounds, minerals). Particularities: Vitamin C is volatile and degrades easily depending on temperature, humidity and oxygen level
	Vitamin E	Acceptable levels of intake are indicated by values of 5–20 μg/mL in adults and children aged 12 years and over, and 3–15 μg/mL for younger children. Toxicity: intakes greater than 900 mg per kg of the diet. Toxicity signs: - headache, nausea, muscle weakness, double vision, and creatinuria, and gastrointestinal disturbances	Forms: Natural vitamin E (d-alpha-tocopherol) is more bioavailable than synthetic vitamin E (dl-alpha-tocopherol) Absorption efficiency: varies significantly, between 10% and 79%. Factors influencing bioavailability: food matrix, fat content, interaction with fat-soluble micronutrients
	B vitamin complex	B1: 0–12 months: 0.3 mg/1,000 kcal 1–50: 0.4 mg/1,000 kcal Toxicity: more than 3 g/d Toxicity signs: - headache, irritability, insomnia, weakness, tachycardia, and pruritis B2: 0–1 years: 0.4 mg/day 1–10 years: 0.6–1 mg/day Males 11–14: 1.2 mg/day Males 15+: 1.3 mg/day Females 11+: 1.1 mg/day Toxicity: low B6: 0–6 months: 8 μg/g protein 7–9 months: 10 μg/g protein 10–12 months: 13 μg/g protein 1–50 years: 15 μg/g protein Toxicity: >500 mg/day Toxicity signs: - peripheral neuropathy and loss of sensation in the feet B9: 0–1 years: 50 μg/day 1–3 years: 70 μg/day 4–6 years: 100 μg/day 7–10 years: 150 μg/day Males 11+ years: 200 μg/day Females 11+ years: 200 μg/day Toxicity: low B12: 0–1 years: 0.3–0.4 μg/day 1–3 years: 0.5 μg/day 4–6 years: 0.8 μg/day 7–10 years: 1 μg/day 11–14 years: 1.2 μg/day Males 15+ years: 1.5 μg/day Females 15+ years: 1.5 μg/day Toxicity: not reported	Various processes, including thermal preparation (boiling, baking, frying) of food can reduce the amount of vitamins in the B complex Natural and synthetic B vitamins had comparable bioavailability Serum levels of each B vitamin following a supplementation period of approximately 6 weeks Thiamine (B₁): +23% (Natural), +27% (Synthetic) Riboflavin (B₂): +14% (Natural), +13% (Synthetic) Pyridoxine (B₆): +101% (Natural), +101% (Synthetic) Folic acid (B₉): +86% (Natural), +153% (Synthetic) Cobalamin (B₁₂): +16% (Natural)
Minerals	Zinc	0–6 months: 4.0 mg/day 7 months–3 years: 5.0 mg/day 4–6 years: 6.5 mg/day 7–10 years: 7.0 mg/day 11–14 years: 9.0 mg/day Males 15+ years: 9.5 mg/day Females 15+ years: 7.0 mg/day Toxicity: 2 g or more	Absorption: intestinal The fractional absorption of dietary zinc in humans is usually in the range of 16%–50%, regulated by zinc homeostasis The bioavailability of zinc from a mixed or vegetarian diet based on refined grains is estimated to be 26%–34%, while 18%–26% is absorbed from a diet based on unrefined grains
	Selenium	0–3 months: 10 mg/day 4–6 months: 13 mg/day 7–12 months: 10 mg/day 1–3 years: 15 mg/day 4–6 years: 20 mg/day 7–10 years: 30 mg/day 11–14 years: 45 mg/day Men 15–18 years: 70 mg/day 19+ years: 75 mg/day Women 15+ years: 60 mg/day Toxicity: >6 μg/kg/zi Toxicity signs: - nausea, vomiting, and fever - intakes of 50 mg of Zn have been shown to interfere with Cu and Fe metabolism	The bioavailability of selenium depends primarily on its chemical form Selenocompounds (both organic and inorganic) play a vital role in improving the bioavailability of selenium Intestinal microflora metabolize these selenocompounds, turning them into selenomethionine, which is then incorporated by intestinal bacteria Assessing selenium status - Biomarkers of intake: these assess food consumption through questionnaires - Biomarkers of retention/excretion: these measure selenium levels in urine, feces, nails, hair and plasma - Biomarkers of selenium functionality: these include GPX3 in plasma as well as GPX1 in erythrocytes, lymphocytes and tissue samples
	Magnesium	0–3 months: 55 mg/day 4–6 months: 60 mg/day 7–9 months: 75 mg/day 10–12 months: 80 mg/day 1–3 years: 85 mg/day 4–6 years: 120 mg/day 7–10 years: 200 mg/day 11–14 years: 280 mg/day 15–18 years: 300 mg/day Toxicity: If renal function is normal hypermagnesaemia is virtually impossible to achieve by dietary means.	There is a difference in bioavailability between different forms of presentation of magnesium (effervescent tablets or granule formulations are more effective than tablets due to their solubility) The percentage of absorption decreases with increasing dose Organic forms are better absorbed than inorganic forms. Magnesium taurate appears to be one of the most bioavailable salts
Polyphenols	Quercetin	There are no reports	Dietary sources of quercetin include lettuce, chili peppers, blueberries, onions, black chokeberry, black elderberry, capers, tomatoes, broccoli, and apples The type of sugar moiety attached to quercetin affects its bioavailability Quercetin is lipophilic and dietary fat increases its bioavailability Indigestible fiber can improve the absorption of quercetin
	Resveratrol	There are no reports	Resveratrol undergoes rapid metabolism in the liver, resulting in low oral bioavailability

Finally, in agreement with the above, we reiterate that the administration of antioxidants in pediatric patients with diabetes involves distinct challenges. In this sense, it is necessary to institute some well-regulated supplementary measures, to avoid the risks of toxicity. Primarily, the clinician must consider the fact that children, mainly those with diabetes, have a particular metabolism. This is partly due to both the underlying disease and the rapid growth stage they are facing. Consequently, they are more susceptible to the accumulation of free radicals. Also, their endogenous antioxidant capacity is still developing, thus there is a marked risk of imbalances. The challenge in this situation is to provide an adequate intake of antioxidants without interfering with the body’s physiological functions. To achieve this, regular monitoring of metabolic status and glycemic levels is recommended. The dosage, frequency and form of administration must be carefully adapted to the age, weight and metabolic status of each child. An obstacle in this initiative is represented by the limitation of the existence of clinical studies investigating the effectiveness and safety of antioxidants (e.g., alpha-lipoic acid, coenzyme Q10 and glutathione) in children. However, certain antioxidant substances such as vitamin A, vitamin E, selenium or natural herbal products are known to be toxic at high doses. They can interfere with liver function, bone balance or even blood clotting. Also, excessive intake of antioxidants can stimulate a strong pro-oxidant effect. The final effect is, contrary to the therapeutic purpose, the production of free radicals or the potentiation of their reactivity. Last but not least, antioxidant supplements can interact with insulin or other antidiabetic drugs, thereby destabilizing the glycemic balance and therapeutic effectiveness. The strategies useful in limiting the overdose of antioxidants are represented by the observance of the recommended doses (where these are known) - mentioned in table 3, as well as the preference towards the promotion of a diet rich in natural antioxidant foods. The periodic evaluation of the individual metabolic response remains indispensable (Franco et al., 2021; Timbo et al., 2006; Timbo et al., 2018; Or et al., 2019; Geller et al., 2015; Navarro and Seeff, 2013; Vogiatzi et al., 2014; Ibrahim et al., 2021).

Another peculiarity of toxicity induced by antioxidants resides in the drug-drug interaction (DDI). Thus, remembering that antioxidants, especially those from a diet rich in polyphenols, can interact to influence the absorption, metabolism, distribution and elimination of certain drugs. The mechanisms targeted by polyphenols are the modulation of the activity of CYP450 liver enzymes (responsible for drug metabolism) or transporter proteins (essential in regulating the bioavailability of drugs). Among the most common examples of drug-antioxidant interactions include:

- Green tea/grapefruit juice can potentiate the activity of statins, antidepressants or anxiolytics by increasing their blood concentration.
- Flavonoids from fruits and vegetables can inhibit P-glycoprotein, leading to the accumulation of some drugs (e.g., protease inhibitors, digoxin, cyclosporine) in the body.
- The antioxidants in green tea and dietary fibers can interfere with the absorption of drugs such as tetracyclines or bisphosphonates, reducing their effect if they are administered simultaneously.
- Vitamin C can influence the absorption of other drugs (e.g., ketoconazole) through its effect on the characteristics of the internal environment - the pH.
- Vitamin E and polyphenols can increase the risk of bleeding if they are administered simultaneously with anticoagulants such as warfarin, whose metabolism rate they can change.
- Antioxidants (e.g., vitamin C) can reduce the effectiveness of chemotherapeutics (e.g., cisplatin) by combating oxidative stress, a mechanism used by pharmaceutical substances to destroy tumor cells.
- In particular, it has been reported that resveratrol and curcumin can cross the blood-brain barrier, modulating the levels of neurotransmitters as well as the effectiveness of antidepressants or anxiolytics (Chang, 2009; Du et al., 2012; Hanley et al., 2011; Zha, 2018; Parasuraman and Maithili, 2014; Saso and Firuzi, 2014; Fan et al., 2017; Heaney et al., 2008; Rendeiro et al., 2015; Wang et al., 2022).

To mitigate the effects of DDI, the medical literature proposes the periodic evaluation of patients following chronic treatments with drugs metabolized by CYP450, especially if they use antioxidant supplements. Thus, regular blood tests are considered to estimate and adjust drug doses. It also emphasizes the importance of educating patients about the potential risks of supplements and antioxidants, especially if they are on a therapeutic regimen that includes drugs considered critical in terms of interactions (Chang, 2009; Saso and Firuzi, 2014; van Roon et al., 2005).

## 6 Conclusion

Diabetes is a multifaceted condition that is primarily distinguished by elevated blood glucose levels. The excessive production of free radicals and oxidative stress are among the most notable consequences of this imbalance. Free radicals above the normal amount interfere with internal homeostasis, causing damage at the cellular level. This is one of the pathogenic ways leading to the emergence of chronic diabetic complications. Consequently, the role of antioxidants in the management of pediatric diabetes (prophylactic/adjuvant) represents an essential aspect of the approach, partly due to the ability to counteract oxidative stress. The key points of the paper are represented by the summary presentation of the interferences between nutrients and the metabolic pathways affected by oxidative stress, as well as by highlighting the main nutrient-rich foods. Consequently, we believe that the present study has succeeded in publicizing the advantages of food antioxidants and pharmacological products in safeguarding cells from injuries. In turn, it contributes to the reduction of morbidity in the medium and long term. Nevertheless, it is crucial to reiterate that the utilization of antioxidants must be conducted with prudence, caution, and the guidance of a physician. Otherwise, inadequate doses or uncontrolled use may have unwanted effects. Thus, the modulation of oxidative stress and diabetic pathology by means of antioxidants represents a topical issue of particular importance both in research and in clinical practice. The primary objective of future perspectives is to reduce the burden of the disease and improve the pharmacological treatment by focusing on the deepening of studies on antioxidants in a variety of inflammatory or autoimmune pathologies, in conjunction with the development of personalized dietary schemes.
